# Supplementary material for: Sera from Patients with NMOSD Reduce the Differentiation Capacity of Precursor Cells in the Central Nervous System
Source: Int J Mol Sci. 2021 May 14;22(10):5192. doi: 10.3390/ijms22105192 (PMC8155872; doi:10.3390/ijms22105192)
Supplement: Supplementary file 1 [file ijms-22-05192-s001.zip › ijms-1178094-supplementary.pdf]

# *Supplementary Material 1.*

Table 1**.** Clinical, diagnostic, and treatment data from patients with NMOSD.

| **Patient** | **Disease progression (years)** | **First event** | **Core clinical characteristics** | **AQP4-IgG** | **Other markers** | **Immunosuppressant treatment** | **MRI findings** |
|---|---|---|---|---|---|---|---|
| **1** | 20 | Myelitis | Acute myelitis | + | **Anti-ANAs** | + | Few brain white matter T2 lesions, cervical and dorsal spinal cord T2 lesions |
| **2** | 10 | Myelitis | Optic neuritis, acute myelitis | + | **None** | + | Few brain white matter T2 lesions, optic nerve T2 lesions, cervical spinal cord T2 lesions |
| **3** | 10 | Myelitis | Acute myelitis | + | **None** | + | Few brain white matter T2 lesions, optic nerve T2 lesions, cervical spinal cord T2 lesions |
| **4** | 15 | Optic neuritis | Optic neuritis | + | **None** | + | Few brain white matter T2 lesions, optic nerve T2 lesions |
| **5** | 8 | Myelitis | Acute myelitis | + | **Anti-ANSs** | + | Cervical and dorsal spinal |

| | | | | | | | |
|---|---|---|---|---|---|---|---|
| | | | | | | | cord T2 lesions |
| **6** | 6 | Progressive myelitis | Progressive myelitis | + | **None** | + | Few brain white matter T2 lesions, optic nerve T2 lesions. cervical spinal cord T2 lesions |

# *Supplementary Material 2.*

**Supplementary data.**

*Confirmation of the presence of AQP4-IgG in the serum samples and mouse cerebella treated with serum.([i])*

We obtained 16 µm cryosections from the cerebella of adult mice, fixed in 4% paraformaldehyde and subsequently incubated in a PTA solution (PBS 0,1 M, GIBCO 10010023; Triton X-100 0,1%, Thermo 85112 and Albumin 0,1% Sigma A9430) with 10% normal goat serum (Abcam ab7481). Samples were subjected to a rapid immunohistochemical test: tissues were washed in PBS for five minutes to remove possible debris of optimum cutting temperature solution (inclusion medium used to transfer tissue sections to the cryostat) that may have remained after tissue fixation; samples were then washed three times with PTA for five minutes each time. Subsequently, each sample was incubated for 30 minutes with serum from a pooled NMOSD patient, or serum from a healthy control, diluted in 0.1 M buffer saline at a concentration of 1:25, the concentration that yielded the best results in laboratory tests performed with different concentrations. Samples were then washed several times with PTA and incubated with a commercial anti-human IgG (whole molecule)−FITC antibody produced in rabbit (Sigma, ref. F4512-1ML); DAPI (Abcam ab228549) was added at a dilution of 1:2,000 to visualize cell nuclei, and DRAQ5™ (Abcam ab108410) was added at a dilution of 1:4,000. The Olympus AF1000 confocal microscope software was then used to measure the degree of fluorescence of each sample. Measurements were taken from 20 12-bit images per tissue, at a wavelength appropriate for the fluorophore (488 nm excitation; absorption/emission = 512 mm/522 nm). The color measurement scale included brightness steps from 0 to 4096 pixels, the maximum saturation value for the color scale in the ImageJ software. We subsequently measured cell labeling intensity only in the specific areas where the AQP4-IgG autoantibody is known to be expressed. These data were obtained by delimiting the areas of interest for expression of the AQP4-IgG autoantibody (mainly astrocytes and around the blood vessels, where pericytes are found (see supply. Mat. 4) also analyzed according to the results obtained) with a confocal microscope using the Olympus AF1000 device software; regions of interest were identified for more detailed analysis.

---

[i] Kim YJ, Jung SW, Kim Y, Park YJ, Han K, Oh EJ. Detection of anti-aquaporin-4 antibodies in neuromyelitis optica: comparison of tissue-based and cell-based indirect immunofluorescence assays and ELISA. J Clin Lab Anal. 2012;26(3):184-189. doi:10.1002/jcla.21508

# *Supplementary Material 3.*

## Supplementary data.

Primary culture of neurospheres from the SVZ.[(i)]

To obtain SVZ cells, we dissected the brains of three adult BALB/c mice of six weeks of age. The primary neurosphere culture was obtained according to the procedure designed by Belenguer et al.[i] We used a papain solution and the following culture medium reagents:

| | ***Reagent stock*** | ***Working conc.*** | ***Conc.(storage T)*** | ***Provider*** | ***Cat. no.*** |
|---|---|---|---|---|---|
| *Preparation of "control medium"* | DMEM/F12 (1:1) with L-Glutamine | 1× | 1x (4ºC) | Gibco, BRL | 11320-074 |
| | D(+)-Glucose | 0.6% | 30% (20ºC) | Panreac | 141341 |
| | $NaHCO_3$ | 0.1% | 7.5% (4ºC) | Biowest | L0680-500 |
| | HEPES | 5 mM | 1 M (4ºC) | Biowest | L0180-100 |
| | L-Glutamine | 2 mM | 200 nM (20ºC) | Gibco, BRL | 25030-081 |
| | Antibiotic/Antimycotic | 1× | 100× (20ºC) | Gibco, BRL | 15240-062 |
| | "Hormone mix" | 1× | 10× (20ºC) | Homemade | |
| | Heparin sodium salt | 0.7 U/mL | 350 U/mL (4ºC) | Sigma | H3149 |
| | Bovine serum albumin (BSA) | 4 mg/mL | Powder (4ºC) | Sigma | B4287 |
| *Preparation of 10X "hormone mix".* | DMEM/F12 (1:1) with L-Glutamine | 1× | 1× (4ºC) | Gibco, BRL | 11320-074 |
| | D(+)-Glucose | 0.6% | 30% (20ºC) | Panreac | 141341 |
| | $NaHCO_3$ | 0.1% | 7.5% (4ºC) | Biowest | L0680-500 |
| | HEPES | 5 mM | 1 M (4ºC) | Biowest | L0180-100 |
| | Apo-Transferrin | 0.8 mg/mL | Powder | Sigma | T2252 |
| | Bovine insulin | 500 nM | 5 µM in 0.01 N HCl | Sigma | I6634 |
| | Putrescine | 0.1 mg/mL | 1 mg/mL | Sigma | P7505 |
| | Progesterone | 0.2 nM | 2 mM in 95% EtOH | Sigma | P6149 |
| | Sodium selenite | 0.3 µM | 3 mM | Sigma | S9133 |
| *Preparation of "complete medium"* | Control medium | Homemade | | | |
| | EGF | 20 ng/mL | 4 mg/mL (20°C) | Gibco, BRL | 53003-018 |
| | bFGF | 10 ng/mL | 25 mg/mL (20°C) | Sigma | F0291 |

DMEM/F12 (Dulbecco's Modified Eagle Medium/Ham's F12 Nutrient Mixture). Mix well all the components adding last the BSA powder. Incubate in a water bath at 37°C until BSA has dissolved; bring to final volume with DMEM/F12; filter, sterilize, and store at 4°C.

Sections from the subventricular zone were then transferred to a tube with 3 mL of papain (1 mL per brain), and incubated at 37°C for 30 minutes; digestion was then stopped by adding 9 mL of control medium. Sections were centrifuged at 100 g and 21°C for two minutes, the supernatant was removed, and 8 mL of control medium was added. They were filtered using a 50 mL Falcon tube, and 3 mL of complete medium were added. Cells were seeded in nine wells of a p12 plate. In each well, 333 µL of the solution of cells and complete medium was added, followed by 1.2 mL of complete medium, to reach a total volume of 1.5 mL. They were incubated at 37°C in a 5% $CO_2$ humidified incubator so that undifferentiated neural cells would separate and begin to form neurospheres. After approximately

seven days, primary neurosphere cultures expand. To amplify the number of cells, we followed the cell passage protocol. A cell passage was performed once per week for four weeks to obtain an adequate number of cells to conduct the experiment. Passage number 5 was chosen to perform the experiments, since we know that the phenotypic signature of the neurospheres remains unchanged until passage number 10 (ICC and WB, data not shown). The cellular confluence observed was 70% for the neurospheres. The ratio of expansion was 1:3-1:5, which is consistent with those reported in the literature, considering this as the expected behavior of healthy cells. Before beginning the experiment, we proceeded with mycoplasma analysis, for which a MycoAlert PLUS detection kit was used according to the manufacturer's protocol, in passage number 5. The content of the wells was transferred to a 50 mL Falcon tube and the plate was washed with 1 mL of control medium per well, which was pooled with the pre-collected cells to maximize cell recovery. It was centrifuged at 120 g for seven minutes. We then added 600 µL of Accutase® solution to the cell pellet obtained and incubated it at 37°C for 10 minutes. Having ensured that cells were well disaggregated, we added 2,400 µL of complete medium to inactivate the Accutase® solution; 9 mL of control medium was added in the first three passages to remove any possible debris. It was centrifuged at 200 g for 10 minutes. The cell pellet was resuspended with 1 mL of complete medium. Cells were then counted in the Neubauer chamber and 10,000 cells/cm$^2$ were seeded in complete medium at room temperature.

[i]Belenguer G, Domingo-Muelas A, Ferrón SR, Morante-Redolat JM, Fariñas I. Isolation, culture and analysis of adult subependymal neural stem cells. Differentiation. 2016;91(4-5):28-41. doi:10.1016/j.diff.2016.01.005

# *Supplementary Material 4.*

Serum from NMOSD patients colocalize IHC with pericyte markers

In the analysis of the immunostaining using the group of sera from patients with NMOSD, expression is observed in astrocytes and cells that apparently could correspond to pericytes. In order to verify this point, a double immunostaining was performed for the specific marking of pericytes, using the NG2 antibody already described in the literature as a pericyte marker (1-3). Commercial antibody against NG2 (MS, NG2 Millipore MAB5384, 1: 200) was used, with which we performed an IHC and subsequently analyzed the samples with a confocal microscope. Olympus AF1000 microscope software, using a sequential acquisition protocol with a resolution of 800 px, at 4 msec x pixel, with a magnification of 60X (NA 0.84); regions of interest were identified for further analysis of co localitation. Image A and B show the colocalization of the antibody with the serum of patients with NMOSD.

Image A

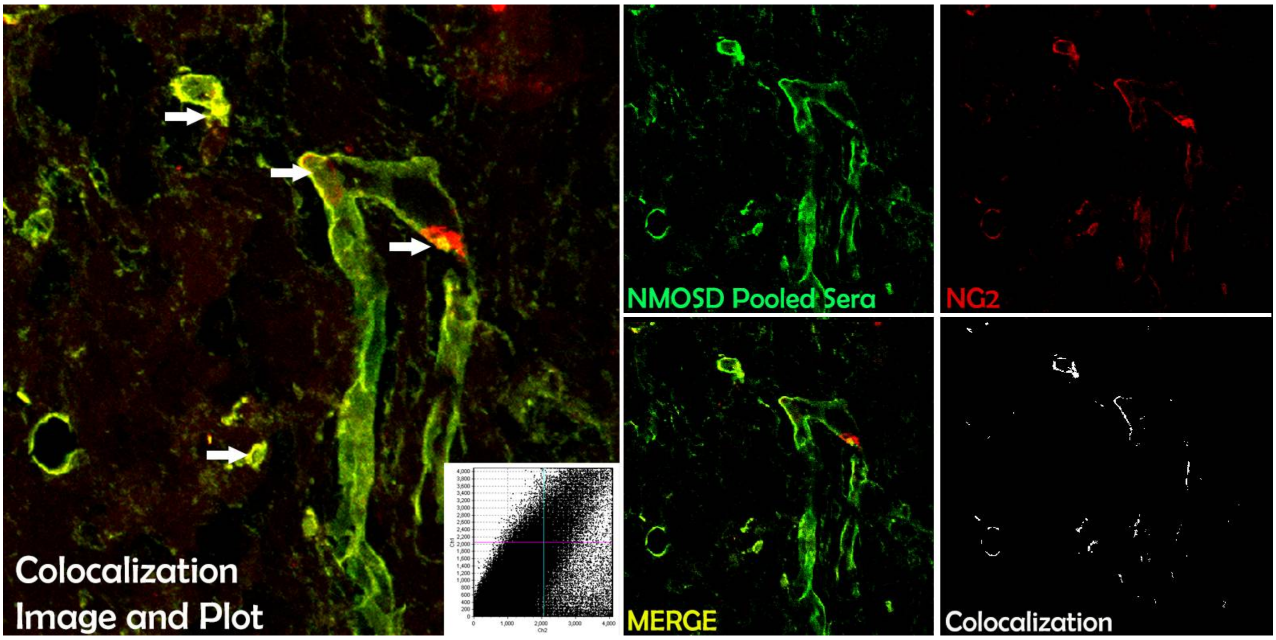


Confocal microscopy image acquired at 60x with a 2x digital zoom, in channel-by-channel mode (sequential mode, 800 x 800 pixels). It shows the fluorescence image and the colocalization plot, in the individual panels we observe in A) channel 488 in green NMOSD Sera, B) in channel 555 in red the ac NG2, C) shows the fusion of both channels (red and green) and in D) the image of the colocalization analysis (white doots). Arrows indicate double marking and colocalization.

Image B

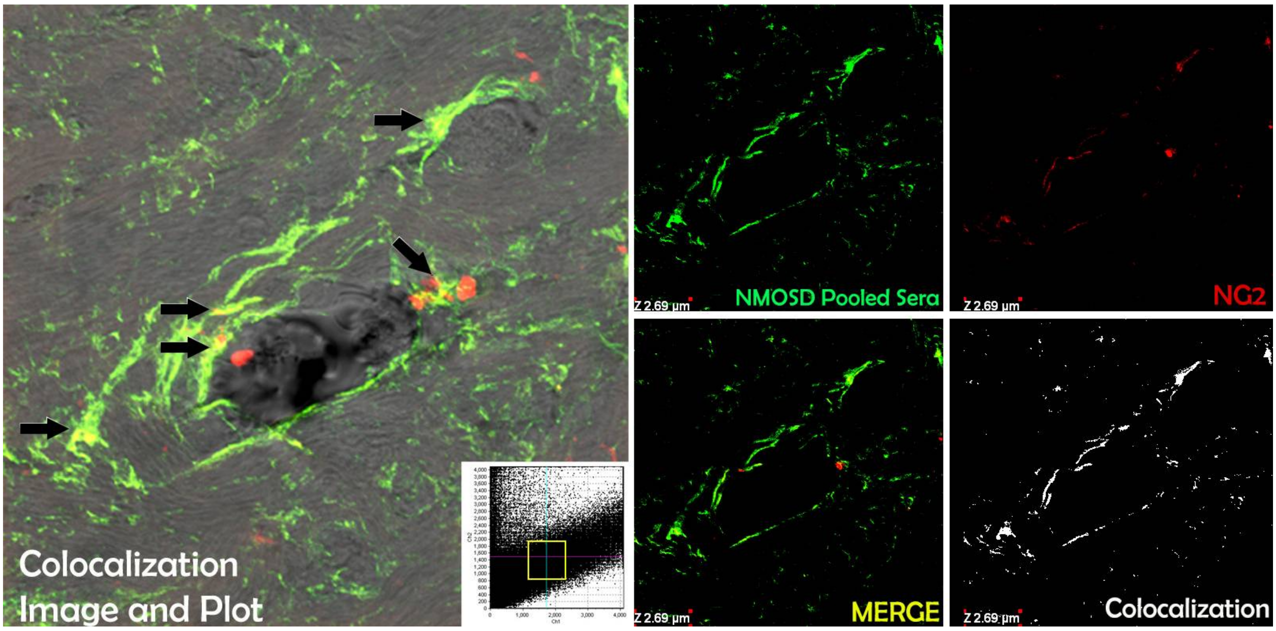


Confocal microscopy image acquired at 60x with a 2x digital zoom, in channel-by-channel mode (sequential mode, 800 x 800 pixels), with transmitted light image as spatial orienting. It shows the fluorescence image and the colocalization graph, in the individual panels we observe in A) channel 488 in green NMOSD Sera, B) in channel 555 in red the ac NG2, C) shows the fusion of both channels (red and green) and in D) the image of the colocalization analysis (white doots). Arrows indicate double marking and colocalization

Referencias

1. Yamazaki Tomoko, Mukouyama Yoh-suke.Tissue Specific Origin, Development, and Pathological Perspectives of Pericytes. Frontiers in Cardiovascular Medicine (2018) 5: 78. doi: 10.3389/fcvm.2018.00078
2. Ozerdem U, Grako KA, Dahlin-Huppe K, Monosov E, Stallcup WB. NG2 proteoglycan is expressed exclusively by mural cells during vascular morphogenesis. Dev Dyn. (2001) 222:218–27. doi: 10.1002/dvdy.1200
3. Stallcup WB. The NG2 proteoglycan: past insights and future prospects. J Neurocytol. (2002) 31:423–35. doi: 10.1023/A:1025731428581
